# Spontaneous preterm birth and cervical length in a pregnant Asian population

**DOI:** 10.1371/journal.pone.0230125

**Published:** 2020-04-13

**Authors:** Serene Thain, George S. H. Yeo, Kenneth Kwek, Bernard Chern, Kok Hian Tan

**Affiliations:** 1 Department of Maternal Fetal Medicine, KK Women’s and Children’s Hospital, Singapore, Singapore; 2 Singapore General Hospital, Singapore, Singapore; 3 Minimally Invasive Surgery Unit, KK Women’s and Children’s Hospital, Singapore, Singapore; Azienda Ospedaliero Universitaria Ospedali Riuniti di Ancona Umberto I G M Lancisi G Salesi, ITALY

## Abstract

**Objective:**

Preterm birth (birth before 37 weeks of completed gestation) is the leading cause of neonatal death, and has an incidence of 5–13% which is believed to be on the rise. The objective of this study was to determine the rate of spontaneous preterm birth and investigate the relationship between preterm birth and cervical length in a pregnant Asian population.

**Materials and methods:**

A prospective observational study between September 2010 and November 2013 was performed at KK Women’s and Children’s Hospital, Singapore. 1013 women with single viable pregnancies were recruited at less than 14 weeks of gestation between September 2010 and November 2013, excluding those with multiple gestation, pre-existing autoimmune or renal disease or those with current pregnancies complicated by aneuploidy or fetal anomalies. Participant characteristics were obtained from an interviewer-administered questionnaire at the first recruitment visit. Cervical length was measured using ultrasound at each of the 4 antenatal visits (Visit 1: < 14 weeks, Visit 2: 18–22 weeks, Visit 3: 28–32 weeks and Visit 4: > 34 weeks) using the Fetal Medicine Foundation protocol. Data on pregnancy outcomes were obtained from obstetric case notes and records. The main outcome measure examined in this study was that of spontaneous preterm birth and its relationship to cervical length.

**Results:**

There was a significantly shorter cervical length both in the 2nd trimester (18 to 22 weeks) and the 3rd trimester (28 to 32 weeks) in the preterm birth group compared to the term birth group (p = 0.028 and p < 0.001 respectively). In the first trimester (11 to 14 weeks), there was no statistically significant difference in cervical length between the two groups (p = 0.425). ROC curve analysis for cervical length in the preterm birth group for 18 to 22 weeks and 28 to 32 weeks showed an AUC of 0.605 and 0.725 respectively. At 28 to 32 weeks of gestation, a cut-off level at 2.49 cm has a sensitivity of 54.8%, specificity of 82.5%, negative predictive value of 97.9% and positive predictive value of 11.1%.

**Conclusion:**

There is a significantly shorter cervical length in the 2nd and 3rd trimester in the preterm birth group. Cervical length is a moderate predictor of preterm birth with good negative predictive value and a relatively good specificity. Ultrasound cervical length screening for pregnant Asian women between 18 and 22 weeks of gestation with a cutoff of ≥ 2.48cm can help to identify a group of women who are at risk for preterm birth.

## Introduction

Preterm birth, defined by WHO in 1977 as all births before 37 completed weeks of gestation or fewer than 259 days since the first day of a woman’s last menstrual period, is the single largest and most commonly cited adverse outcome in pregnancy [1]. Reported incidence for preterm births ranges from 5–13%, and this number is believed to be on the rise [2,3]. In Singapore, about 3500, or 1 in 11 (out of 39615) babies, were born prematurely in 2017. [4] Of all these preterm births, spontaneous preterm labour accounts for about 50% of them.

The increasing trend of preterm births is alarming, as over 1 million children die each year due to complications of preterm birth. Prematurity is the leading cause of neonatal death and the 2^nd^ leading cause of death after pneumonia in children under the age of 5. In our tertiary centre, over a period of 10 years from 2001 to 2010, prematurity was found to be the largest contributor to neonatal mortality at 47.5%. Also, significant morbidities to the child can follow the event of a preterm birth. Babies born a few weeks early are 6 times more likely to die in their first week of life than full-term babies, and 3 times more likely to die before their first birthday [5]. Not only that, many survivors of preterm birth face an increased risk for neonatal health complications and lasting disabilities such as mental retardation, cerebral palsy, lung and gastrointestinal problems as well as vision and hearing loss.

The etiology of preterm labour and delivery is one that is still of great research interest worldwide. It has been widely hypothesized that preterm labour and delivery is a syndrome initiated by multiple mechanisms, from mechanical factors (such as uterine overdistension), inflammation (e.g. infection), circulatory disturbances (e.g. uteroplacental ischemia) or from a combination of several factors [2,6]. The list of clinical risk factors is well known, however, these have been shown to have limited clinical utility. All of the clinical risk factors identified have a low isolated or combined predictive value, and the cause of spontaneous preterm labour remains unidentified in up to half of all cases [7].

A shortened cervical length has been used as one of the major risk factors for a preterm delivery. Identification of women with a short cervix and treatment with vaginal progesterone can reduce the frequency of preterm birth. A recent 2016 systematic review and meta-analysis of randomized trials concluded that vaginal progesterone treatment of asymptomatic women with a short cervix reduced the frequency of preterm birth by 35 percent and reduced composite neonatal morbidity and mortality by 40 percent [8]. It is thus important to ascertain for various populations at what cervical length does the risk of preterm birth increase significantly, such that interventions can be undertaken to minimize the risk of this serious obstetric complication.

Currently, although there have been many studies performed regarding cervical length and preterm birth, these have largely been conducted in the western population. We thus lack information about whether these cervical length measurements apply to an Asian population. The aim of our study was thus to determine the rate of spontaneous preterm birth and to analyse the distribution of cervical length and the relationship between preterm birth and cervical length in the pregnant Asian population here in Singapore.

## Material and methods

The study was approved by the Singhealth Centralised Institutional Review Board [CIRB Ref 2010/214/D]. Written informed consent was obtained from all participants. The study was conducted as part of the Neonatal and Obstetrics Risk Assessment (NORA) prospective cohort study at KK Women’s and Children’s Hospital in Singapore and funded by the National Medical Research Council Programme Project Grant [NMRC/PPG/KKH/2010].

Participants were women with single viable pregnancies recruited at less than 14-week gestation between September 2010 and November 2013. Exclusion criteria included that of multiple gestation, patients with pre-existing autoimmune or renal disease and current pregnancies complicated by aneuploidy or fetal anomalies. These women were followed up from recruitment until their postnatal discharge from the hospital. A total of 4 antenatal visits were included as part of this study, with Visit 1 at less than 14+ 0 week of gestation, Visit 2 at 18 + 0 to 22 + 0 week gestation, Visit 3 at 28 + 0 to 32 + 0 week gestation, and Visit 4 at 34 week gestation and above. Gestational age was determined based on a first trimester dating scan measuring the fetal crown-rump length. Measurements of cervical length were conducted at each of the 4 visits for all patients based on the Fetal Medicine Foundation protocol using a 5MHz transvaginal probe. All sonographers performing the scan had extensive training and accreditation by the Fetal Medicine Foundation for competency in performing this scan. Each examination was performed over a period of 3 minutes and the best shortest measurements of cervical length was recorded.

Participant characteristics, including details of maternal age, race, height, weight, socio-economic status, smoking and alcohol status, medical and surgical history, previous and current obstetric history were obtained from an interviewer-administered questionnaire at the first recruitment visit and entered into a computer database.

Data on pregnancy outcomes were collected from obstetric case notes and records. The primary outcome of interest was that of spontaneous preterm birth before 37 weeks of gestation. The obstetric records of all patients delivering preterm before 37 weeks (<259 days) were examined to determine if the preterm birth was iatrogenic or spontaneous. Spontaneous preterm birth included those with spontaneous onset of labour and those with preterm pre-labour rupture of membranes.

Statistical analyses were performed using Microsoft Excel and SPSS (Version 19). Continuous variables were summarized by the median values, and comparison between groups were conducted using the Mann-Whitney U-test. Univariable comparisons of dichotomous data were performed using Chi-square test and Fisher’s test where appropriate. Statistical significance was set at a p-value of <0.05. Relationship of various variables such as maternal obstetric history and demographic characteristics to that of cervical length were examined for significance. Receiver operating curves were generated at various time points in the pregnancy, and sensitivity/specificity analyses conducted to determine the best cut-off value for cervical length as a predictor for preterm birth, based on the prevalence of preterm birth determined within this study.

## Results

### Study population

A total of 3271 patients were screened for eligibility for inclusion into the study between September 2010 to November 2013. Of these, 2820 patients were deemed eligible for inclusion, and 1013 of them were recruited into the study. 934 (92.2%) of patients completed all 4 antenatal follow-up visits as described above, with 66 (6.5%) patients being lost to follow up and 13 (1.3%) patients who miscarried before 24 weeks of gestation. Of the 934 patients, 17 delivered at other institutions, but delivery data for 9 of them were retrieved via phone call. There were 926 patients in total with available study outcomes that we based our analysis of the data on. The predictive statistics generated later in this paper relating to preterm birth were calculated only from the group of patients who had spontaneous preterm births. Patients who had iatrogenic preterm birth due to other factors such as pre-eclampsia or medical indications were analysed as part of the group without spontaneous preterm birth.

**Fig 1:**
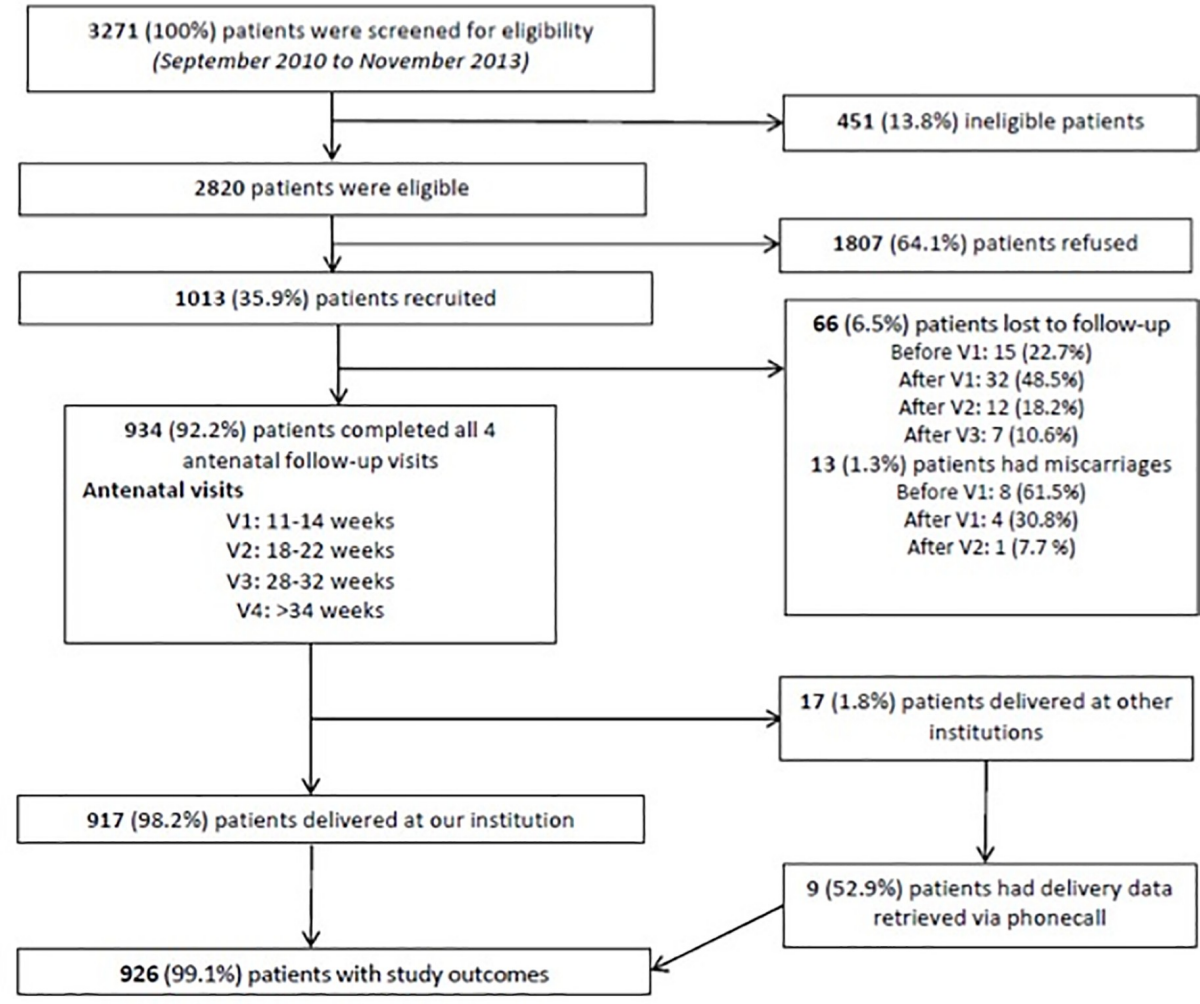
Flowchart showing the NORA study recruitment process.

Fig 1 shows the flowchart demonstrating the recruitment process of the NORA study. Demographics, clinical characteristics of the cohort and pregnancy outcomes are presented in Table 1.

**Table 1:** Demographics, clinical characteristics and pregnancy outcomes of study population.

Characteristics	Incidence (total n = 926) N, %
**Demographics**	
**Age**	
≤ 20	21 (2.27%)
21–25	119 (12.85%)
26–30	336 (36.29%)
31–35	289 (31.21%)
36–40	140 (15.11%)
41–45	21 (2.27%)
**Race**	
Chinese	470 (50.75%)
Malay	250 (27.00%)
Indian	100 (10.80%)
Others	106 (11.45%)
**BMI**	
<18.5	62 (6.70%)
18.5 to 24.9	523 (56.48%)
25 to 29.9	230 (24.84%)
≥30	106 (11.45%)
Unknown	5 (0.54%)
**Education**	
Primary	12 (1.3%)
Secondary	209 (22.57%)
ITE	99 (10.69%)
JC/Polytechnic	268 (28.94%)
University	335 (36.18%)
Unknown	3 (0.32%)
**Marital status**	
Married	871 (94.06%)
Single	52 (5.62%)
Divorced/Widowed	3 (0.32%)
**Employment status**	
Employed	740 (79.91%)
Housewife	176 (19.01%)
Student Unemployed	7 (0.76%) 3 (0.32%)
**Obstetric history**	
**Gravida**	
Primip	389 (42.01%)
Multip	537 (57.99%)
**Parity**	
0	501 (54.10%)
1	295 (31.86%)
2	92 (9.94%)
3	30 (3.24%)
4	5 (0.54%)
5	3 (0.32%)
**Smoking/alcohol intake**	
**Smoking**	
Non-smoker	902 (97.40%)
Smoker	24 (2.60%)
**Alcohol**	
Drinker	915 (98.80%)
Non-drinker	11 (1.20%)
**Pregnancy Outcomes (Gestation at delivery)**	
**Spontaneous Preterm Birth**	39 (4.2%)
< 28 weeks (extreme preterm)	2 (5.1%)
28-<32 weeks (very preterm)	1 (2.6%)
32-<35 weeks (moderate preterm)	7 (17.9%)
35–37 weeks (late preterm)	29 (74.4%)
**Iatrogenic preterm birth**	23 (2.5%)
**Term births**	864 (93.3%)

Of the 926 patients with all 4 completed visits, 62 (6.7%) of patients delivered prematurely at less than 37-week gestation. Of these, 39 (62.9%) of them were spontaneous preterm birth, while 23 (37.1%) of them were prematurity secondary to iatrogenic interventions. Of the spontaneous preterm birth group, 26 (66.7%) of them underwent spontaneous onset of labour, while 13 (33.3%) were associated with premature pre-labour rupture of membranes (PPROM). The prevalence of spontaneous preterm birth in the study population was 4.2%.

**Fig 2:**
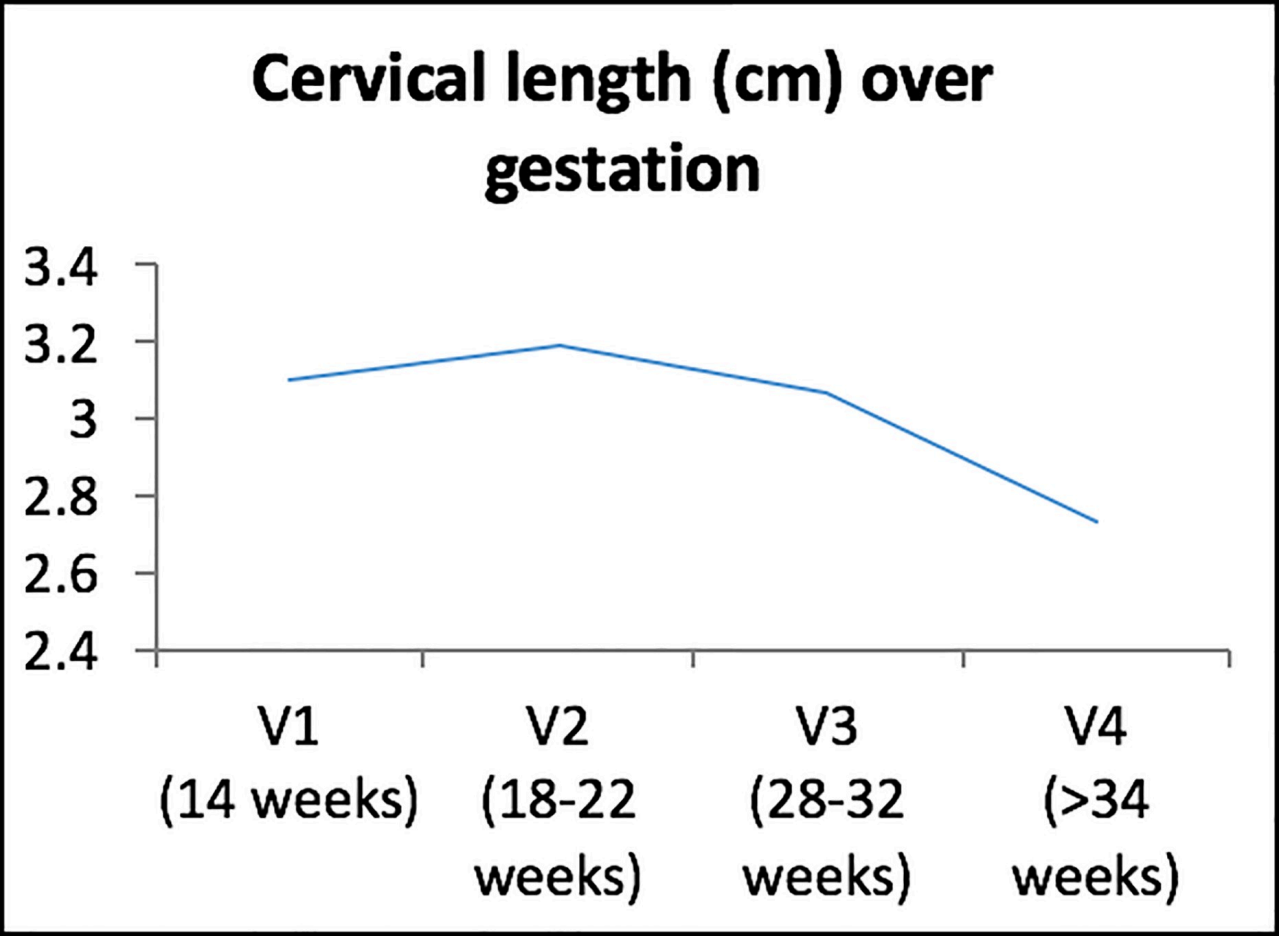
Median cervical length over gestation.

Table 2 shows the median cervical length for patients in the study at various gestations. Fig 2 below depicts this in a graphical representation. It is observed that there is a general decreasing trend of cervical length over gestation especially from the 2^nd^ trimester onwards after 22 weeks.

**Table 2:** Median cervical length over gestation.

	Median cervical length (cm) (n = 911)
**V1 (14 weeks)**	3.10 (± 0.63)
**V2 (18–22 weeks)**	3.19 (± 0.75)
**V3 (28–32 weeks)**	3.07 (± 0.73)
**V4**Ɨ **(>34 weeks)**	2.73 (± 0.82)

Ɨ only includes patients whose gestation at Visit 4 is ≤36 weeks

Table 3 shows the relationship between ethnicity and median cervical length over 4 time points in the pregnancy. At V2 (18–22 weeks) and V3 (28–32 weeks), there was a significant difference in median cervical length observed between the various ethnicities, largely contributed by the shorter median cervical length observed in the Indian population compared to the other 3 groups.

**Table 3:** Relationship between ethnicity and median cervical length.

	Chinese (n = 462)	Malay (n = 246)	Indian (n = 99)	Others (n = 104)	p-value
**V1 (14 weeks)**	3.13 (± 0.65)	3.03 (± 0.61)	2.99 (± 0.67)	3.21 (± 0.58)	0.118
**V2 (18–22 weeks)**	3.22 (± 0.77)	3.13 (± 0.69)	2.97 (± 0.70)	3.33 (± 0.82)	0.009*
**V3 (28–32 weeks)**	3.11 (± 0.74)	2.95 (± 0.71)	2.94(± 0.62)	3.13 (± 0.79)	0.045*
**V4**Ɨ **(>34 weeks)**	2.75 (± 0.82)	2.67(± 0.81)	2.59 (± 0.85)	2.68 (± 0.86)	0.783

Ɨ only includes patients whose gestation at Visit 4 is ≤36 weeks

*significant at p<0.05, Mann Whitney test

ns = not significant

Table 4 demonstrates the median cervical length in patients with a history of previous preterm delivery (n = 33) versus those without (n = 878). There was no significant difference between these 2 groups across all gestations, although there appeared to be a trend towards significance at V2 and V3, with the patients in the previous preterm delivery group having a trend towards a shorter cervical length compared to those with no previous preterm delivery.

**Table 4:** Relationship between median cervical length and history of previous preterm birth.

	Previous Preterm (n = 33)	No Previous Preterm (n = 878)	p-value
**V1 (14 weeks)**	3.01 (± 0.66)	3.10 (± 0.63)	0.835
**V2 (18–22 weeks)**	2.92 (± 0.89)	3.20 (± 0.75)	0.050
**V3 (28–32 weeks)**	2.76 (± 0.85)	3.08 (± 0.72)	0.060
**V4** Ɨ **(>34 weeks)**	2.66 (± 0.81)	2.73 (± 0.82)	0.565

Ɨ only includes patients whose gestation at Visit 4 is ≤36 weeks

*significant at p<0.05, Mann Whitney test

ns = not significant

Table 5 shows a comparison between the median cervical length in patients who eventually had a spontaneous preterm birth in their current pregnancy versus those who delivered at term. There was a significant difference in median cervical length between these 2 groups, with the spontaneous preterm birth group having a shorter median cervical length at V2 and V3 compared to the term birth group.

**Table 5:** Comparison between median cervical length in spontaneous preterm birth versus term birth group.

Median cervical length (cm)			
	Spontaneous PTB	Term birth	p-value
**V1 (14 weeks)**	3.05 (±0.50)	3.10 (±0.64)	.425
**V2 (18–22 weeks)**	3.03 (±1.34)	3.20 (±0.71)	.028*
**V3 (28–32 weeks)**	2.43 (±0.88)	3.10 (±0.71)	< .001*
**V4** Ɨ **(>34 weeks)**	2.21 (±0.73)	2.74 (±0.82)	.072

Ɨ only includes patients whose gestation at Visit 4 is ≤36 weeks

*significant at p<0.05, Mann Whitney test

**Fig 3:**
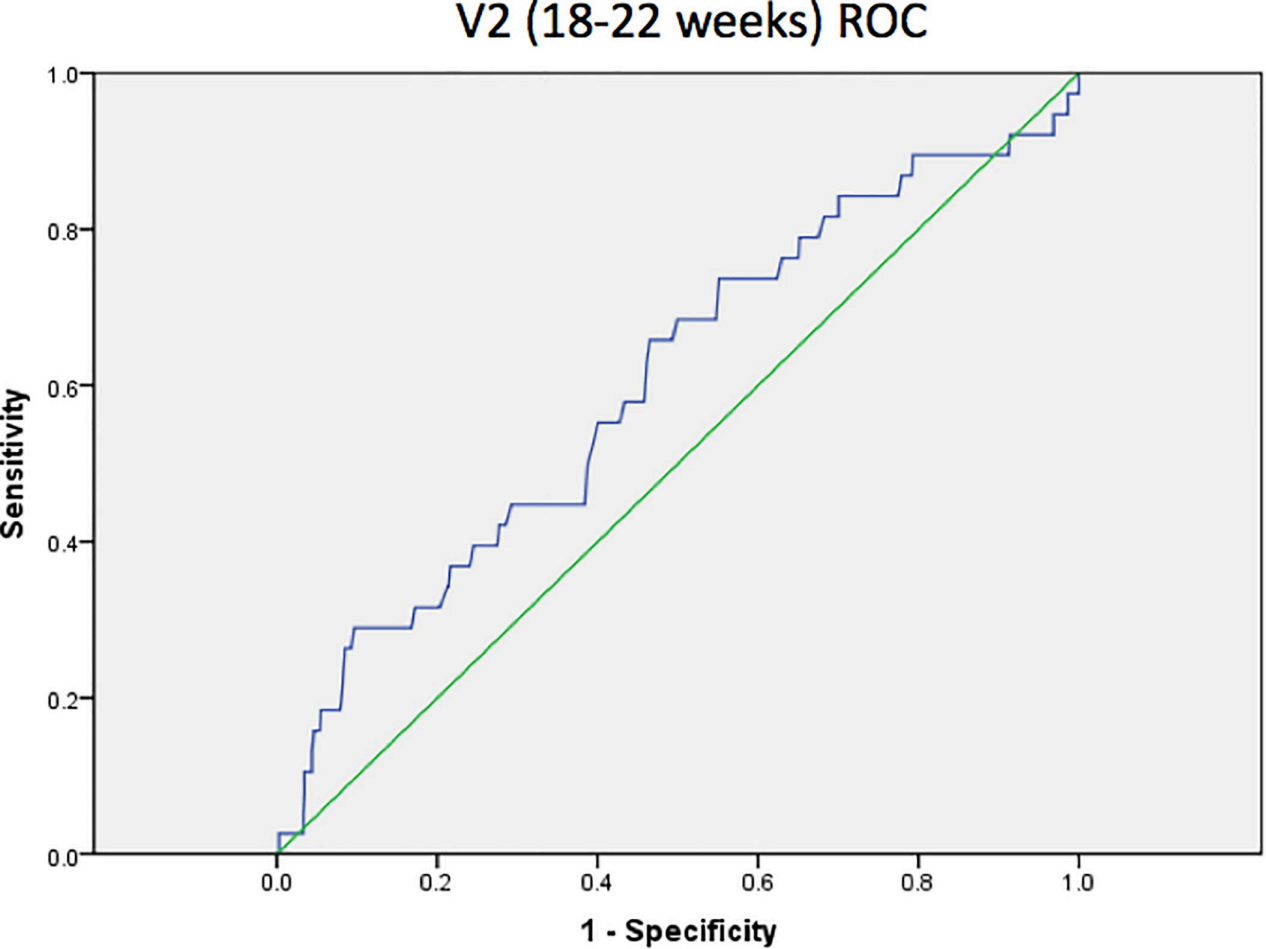
ROC curve for cervical length as a predictor of preterm birth at 18 to 22 weeks’ gestation.

**Fig 4:**
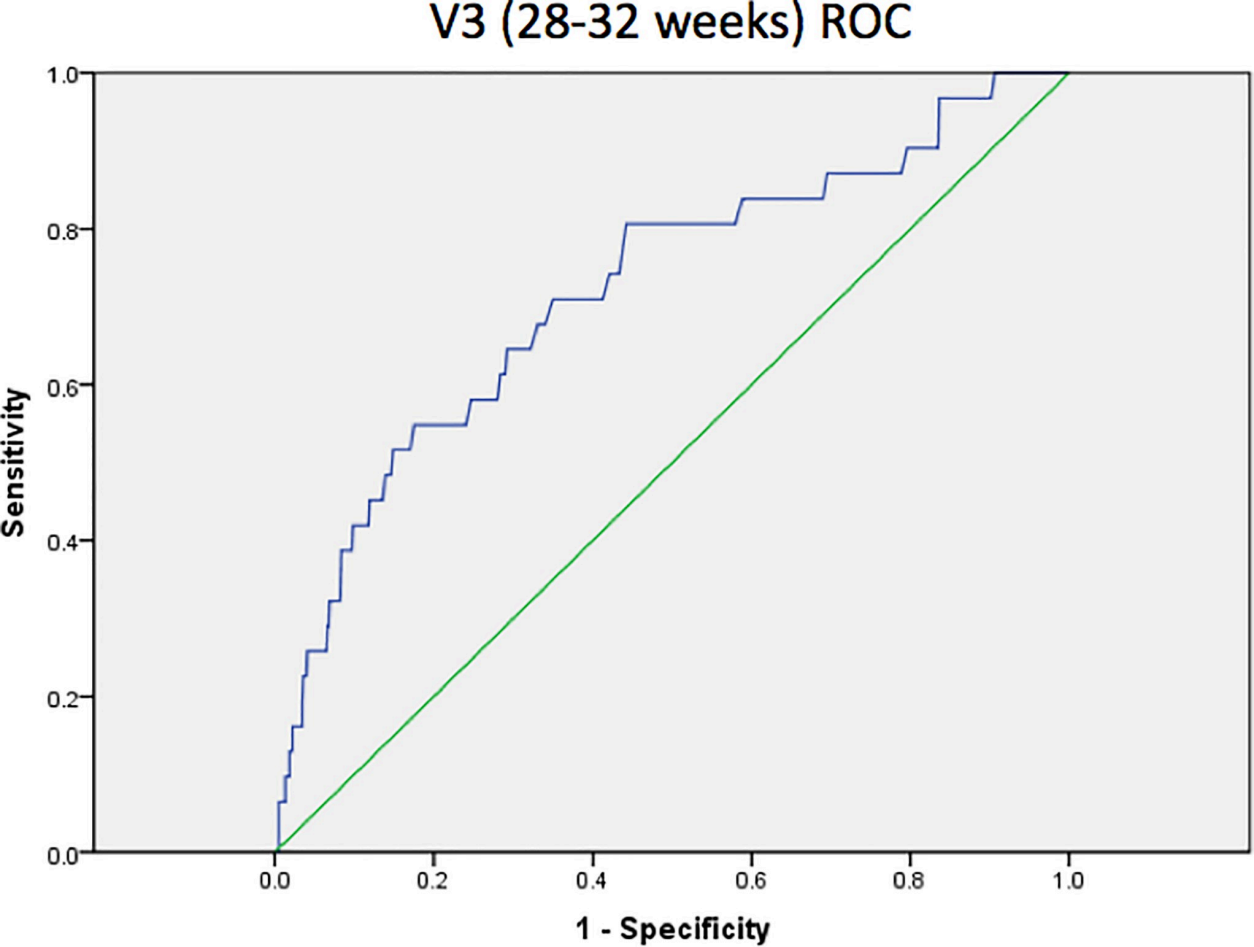
ROC curve for cervical length as a predictor of preterm birth at 28 to 32 weeks’ gestation.

On ROC curve analysis, cervical length showed an area under the curve of 0.61 for the prediction of preterm birth < 37 weeks’ gestation at 18 to 22 weeks (Fig 3). After conducting sensitivity/specificity analyses, the optimal cut-off value was determined to be 2.48cm (sensitivity 29.0%, specificity 90.1%, PPV 12.0%, NPV 96.5%). At 28 to 32 weeks, cervical length showed an improved area under the curve of 0.73 for the prediction of preterm birth < 37 weeks’ gestation (Fig 4). The optimal cut-off value was 2.49cm (sensitivity 54.8%, specificity 82.5%, PPV 11.1%, NPV 97.9%).

## Discussion

Preterm birth is a condition that varies widely between different countries or ethnicities. Reported incidence for preterm births ranges from 5–13%, and this number is believed to be on the rise [2,3]. The national rate of preterm births has also gone up, from 7.2% to 9.5% over the past decade [9]. In this study, the prevalence of spontaneous preterm birth in the study population was 4.2%. This is lower than the national average, but can be attributed to the study population being a selected group of asymptomatic low-risk patients who had fulfilled certain criteria for inclusion into the study, and is therefore not entirely representative of the whole population. However, the rate of 4.2% even in such a low risk group does further emphasize the magnitude of the problem of preterm birth and its implications.

Currently, although there have been several published studies looking at the relationship between cervical length and preterm birth, these studies have largely been conducted in Western/Caucasian populations. However, there is still generally a paucity of data in Asian populations with regards to the clinical question of cervical length and its relationship to preterm birth. We know from existing published studies that the natural history of cervical length across pregnancy is that it is normally distributed and remains relatively constant in pregnancy until the third trimester [10–12]. This was observed similarly in our study population, with the median cervical length remaining relatively constant at V1 and V2 (till 22 weeks), subsequently demonstrating a decline from 28 weeks to 32 weeks and even further past 34 weeks.

It is widely known that cervical length can vary amongst different ethnicities. Perhaps the most common example would be that of African Americans having a significant shorter cervical length compared to Caucasians, with this being been reported in various published studies [13,14]. Studies also previously demonstrated that cervical lengths in pregnant Asian women are different from that of Western women [12,15–17]. This has been postulated to be possibly related to body weight, height or race [15]. We found that ethnicity within our population was also a factor influencing median cervical length, with the Indian population having a significantly shorter median cervical length at V2 (28–22 weeks) and V3 (28–32 weeks) compared to the other ethnicities (Chinese, Malays, Others). Interestingly, the median cervical length in our Indian population appeared shorter compared to other published nomograms in the literature from populations in the Indian subcontinent. A study by Mukherji et al. in 2011 looked at normative data of cervical length in singleton pregnancies of 224 women attending a tertiary care hospital located in eastern India [18]. In this study, cervical length at 20 and 34 weeks was 40.5 ± 1.14 mm (mean ± SD) and 34.8 ± 1.34 mm respectively. It may therefore be useful to generate nomograms for cervical length for each of the various ethnicities specific to a geographic location in view of these inherent differences between continents. Also, more research needs to be conducted with regards to why this variation exists, so as to potentially find interventions that target the underlying contributors to this finding to reduce the rate of preterm birth.

Cervical length is known to be a better predictor of preterm birth in women at increased risk, such as those with a history of spontaneous preterm birth than in asymptomatic women at low risk, and this has been illustrated in multiple studies [19–25]. These studies are usually conducted before 24 weeks’ gestation. Similarly in our study, we saw a significant difference in cervical length between the group with previous preterm delivery and the group without a previous preterm delivery with the spontaneous preterm birth group having a shorter median cervical length at V2 and V3 compared to the term birth group^.^

Although cervical length has been found to be inversely related to the risk of preterm birth in asymptomatic women [20, 26–31], routine screening of cervical length as a predictor of preterm birth in this population is not recommended because of the low incidence of preterm birth in this low-risk population, leading to relatively low positive predictive values. Davies et al. in a Canadian, prospective, blinded observational trial of 964 women (general obstetrical population), found a sensitivity of 57% and a specificity of 82% for preterm birth, using a 30 mm cut-off at 24 to 28 weeks [32]. The positive predictive value for preterm birth (<35 weeks) was only 4.5%, because preterm birth was infrequent. Results of our study show a significantly shorter cervical length in the 2^nd^ and 3^rd^ trimester in the preterm birth group, as consistent with other published studies, with a recommended cut-off value of 2.48cm at 18 to 22 weeks with an area under the curve of 0.61 and 2.49cm at 28 to 32 weeks with an area under the curve of 0.725 according to the ROC curve analysis. Similarly, our study observed that the positive predictive values when using cervical length as a predictor of preterm birth were relatively low at 12.0% and 11.1% at 18 to 22 weeks and 28 to 32 weeks respectively. However, the ROC curve analyses conducted showed cervical length to be a moderate predictor of preterm birth, with a specificity of 90.1% and good negative predictive value of 96.5% at 18 to 22 weeks. This suggests that while cervical length cannot be used to diagnose or confirm at-risk patients, it has a good negative predictive value and relatively good specificity to identify the lower risk patients for preterm birth. By performing cervical length screening at 18 to 22 weeks’ gestation to identify patients at lower risk of preterm birth, we may then be able to identify a separate group requiring closer follow-up or intervention for the rest of the pregnancy. However, the cost-effectiveness of this strategy, which varies with the prevalence of preterm birth in our population, needs to be examined in future studies to determine if this would be economically feasible, balanced against the degree of benefit of potential interventions for preterm birth.

Several limitations need to be acknowledged in our study. Firstly, our study was a prospective study and not a randomized control trial, hence individual clinicians were not blinded to the results of the cervical length measurements performed at each time point in the pregnancy. This resulted in some women receiving prophylactic treatment for short cervices in the form of either oral, vaginal or intramuscular progesterone. We acknowledge that this may have affected the results in the number of spontaneous preterm birth, however it would have been unethical to not allow clinicians to offer progesterone treatment on identification of a short cervix, especially when there is already existing evidence to show that this treatment may be effective in preventing spontaneous preterm birth. In any case, the number of women in our study receiving some form of progesterone therapy during their pregnancy for preterm birth prevention was small (n = 30). Secondly, our patient population was not restricted to that of a low-risk population although this still made up the majority of the group– 33 patients had a history of a previous preterm birth and would hence be classified under the high risk group for spontaneous preterm birth. Including these 33 high risk patients in the ROC curve analysis may affect the cut-off value generated in our study, but these numbers were small compared to the significantly larger majority of low-risk patients.

## Conclusion

In summary, this study shows that in asymptomatic Asian women with a singleton pregnancy, measurement of 2^nd^ trimester cervical length, using a cut-off value of 2.48cm (rounded up to 2.5cm), similar to that in other Western studies, can be useful to identify a group of women who are at risk for preterm birth. These women could potentially undergo increased monitoring and interventions to improve perinatal outcomes.

## Supporting information

S1 Data (XLTX)
